# *Candida albicans* infection inhibits macrophage cell division and proliferation

**DOI:** 10.1016/j.fgb.2012.05.007

**Published:** 2012-09

**Authors:** Leanne E. Lewis, Judith M. Bain, Christina Lowes, Neil A.R. Gow, Lars-Peter Erwig

**Affiliations:** a Division of Applied Medicine, University of Aberdeen, Aberdeen AB25 2ZD, UK; b Aberdeen Fungal Group, University of Aberdeen, Aberdeen AB25 2ZD, UK

**Keywords:** *Candida albicans*, Macrophage, Phagocytosis, Mitosis, Innate immunology

## Abstract

The pathogenicity of the opportunistic human fungal pathogen *Candida albicans* depends on its ability to inhibit effective destruction by host phagocytes. Using live cell video microscopy, we show here for the first time that *C. albicans* inhibits cell division in macrophages undergoing mitosis. Inhibition of macrophage cell division is dependent on the ability of *C. albicans* to form hyphae, as it is rarely observed following phagocytosis of UV-killed or morphogenesis-defective mutant *Candida.* Interestingly, failed cell division following phagocytosis of hyphal *C. albicans* is surprisingly common, and leads to the formation of large multinuclear macrophages. This raises question as to whether inhibition of macrophage cell division is another virulence attribute of *C. albicans* or enables host macrophages to contain the pathogen.

## Highlights

► First demonstration of post-mitotic fusion of macrophages infected with *Candida albicans.* ► Post-mitotic fusion was attributed to *C. albicans* hyphae but not yeast form cells. ► Post-mitotic fusion may inhibit macrophage proliferation and the formation of new uninfected phagocytes.

## 1 Introduction

*Candida albicans* is a major life-threatening human fungal pathogen. Host defence against systemic infection relies mainly on phagocytosis of *Candida* by cells of the innate immune system (Gow et al., 2012). Our recent work has studied *C. albicans*-macrophage interactions using phagocytosis assays and live cell video microscopy coupled with sophisticated image analysis tools (McKenzie et al., 2010; Lewis et al., 2012). Such studies reveal dynamic aspects of the host-pathogen interaction that are not evident from studies based on fixed time point analyses. We have shown, for example, that *C. albicans* can rarely use non-lytic expulsion as a means to escape from macrophages, leaving both pathogen and host cell viable (Bain et al., 2012) and provided detailed information on how hyphal formation within macrophages leads to macrophage lysis and *C. albicans* escape (McKenzie et al., 2010). Here we show that infection of macrophages with *C. albicans* can result in failure of macrophages to complete mitosis. Instead of separating into two individual cells, daughter phagocytes remain together and can fuse to reform a single large macrophage. This intriguing and surprisingly common phenomenon may represent yet another virulence attribute of *C. albicans.*

## 2 Description of observation

Live cell video microscopy was used to study the dynamics of macrophage mitosis. Mitosis of J774.1 macrophages was observed frequently in the absence of *C. albicans.* During the 6 h observation period 30.8 ± 5.2% (mean ± s.e.) of macrophages underwent mitosis, and of these only 0.9 ± 0.9% failed to divide into two separate daughter cells. Video 1 shows an example of a J774.1 macrophage that successfully underwent mitosis. When macrophages were cultured with live *C. albicans*, 29.5 ± 5.7% of macrophages initiated mitosis; however, 35.9 ± 6.1% of 147 mitosis events examined resulted in failed cell division. In Video 2 the macrophage ingests multiple *C. albicans*, which form hyphae within the macrophage. The host macrophage initiated mitosis, but instead of completely separating, the daughter cells remained fused together. In all cases of failed cell separation, *C. albicans* hyphae were observed spanning both daughter cells. Macrophages did not persist in their attempt to undergo mitosis indefinitely. Instead macrophages fused back together on average 44.6 ± 3.3 min after initiation of cytokinesis. It is noteworthy that phagocytosis could still occur whilst macrophages were attempting to undergo mitosis.

Macrophages infected with the *C. albicans* glycosylation mutants, *mnt1Δmnt2Δ*, *mns1Δ* and *mnn4Δ* also underwent frequent post-mitotic fusion. For example, 26.5 ± 3.2% of 134 mitosis events examined failed to complete cell division when J774.1s were cultured with the *mnt1Δmnt2Δ O-*glycosylation mutant In contrast, when macrophages were cultured with UV-killed *C. albicans* 23.8% of macrophages underwent mitosis and of these, only 0.7 ± 0.4% of 143 attempts examined resulted in failed cell division. Mitosis was always successful when J774.1 macrophages were cultured with the hyphal morphogenetic-defective mutant *efg1Δ*. The majority of failed cell divisions observed during this study involved phagocytes that had taken up hyphal *C. albicans* cells, although it was also rarely seen in macrophages infected with clumps of *C. albicans* in the yeast phase morphology.

Mitotically active macrophages were often clustered in specific regions of cultures. Video 3 shows an example of four macrophages in close proximity failing to divide after initiating mitosis. One of the macrophages (B) is ruptured prior to completion of mitosis, whereas three other macrophages initiated mitosis, but the daughter cells remained fused together rather than separating into individual cells. All examples of post-mitotic fusion of daughter cells shown in Video 3 involved macrophages infected with hyphal *C. albicans*.

## 3 Discussion

This is the first report of inhibition of cell division in macrophages cultured with *C. albicans* to date. Inhibition of cell division has previously been reported with the human fungal pathogens *Cryptococcus neoformans* and *Candida krusei (*Luo et al., 2008; García-Rodas et al., 2011). Interestingly, infection with *C. neoformans* has also been shown to promote cell cycle progression as a strategy to spread infection (Luo et al., 2012). Failure to complete cell division in macrophages infected with hyphal *C. albicans* may occur due to difficulties in dividing large cargos, such as hyphae, between daughter cells and our observations suggest that hyphal size may be an important factor in this process. *C. albicans* hyphae may impede spindle formation or prevent the actin ring from contracting and pinching the macrophage into separate daughter cells during cytokinesis. Some microbial pathogens are known to interfere with cell cycle progression, including *Clamydia trachomatis*, which can selectively block cytokinesis (Greene and Zhong, 2003). Likewise, *C. albicans* may either have evolved a specific mechanism to prevent macrophage replication or interfere with this process indirectly due to the enlargement and distension of the phagosome. The observation that *C. albicans* hyphae spanned both daughter cells in all cases of failed cell separation implies mechanical rather than active inhibition of cytokinesis. Interestingly, non-lytic expulsion of hyphal *C. albicans* has been observed immediately prior to a host macrophage undergoing mitosis (Bain et al., 2012). It is possible that, in this instance, expulsion of *C. albicans* may have enabled mitosis and cytokinesis to proceed normally.

Macrophages are pivotal components of the innate immune response to infection with *C. albicans* and mitosis of tissue-derived macrophages plays an important role in macrophage proliferation in infected tissues. Failure of cell division following mitosis induction may inhibit macrophage proliferation and the formation of new uninfected macrophages. Thus, the ability of *C. albicans* to impede daughter cell separation may be yet another mechanism employed by *C. albicans* to aid its survival. An alternative explanation is that this process benefits the host, in that macrophages may sense that they carry dividing fungal cells and inhibit division to prevent spreading of the infection. Interestingly, we observed failed cell division most frequently in clusters of macrophages exposed to and infected with multiple *C. albicans* hyphae. One may speculate that this may result in macrophage giant cell formation that may help suppress growth of large sized microbial cells. Failed cell division is likely to have profound consequences on the host response to infection with *C. albicans* and future studies are needed to establish the mechanism by which phagocytosis of fungal cells inhibits macrophage mitosis.

## 4 Methods

*C. albicans* strain CAI4-CIp10 (NGY152) was grown in SC-Ura medium at 30^o^C, as described previously (Lewis et al., 2012). For phagocytosis assays (McPhillips and Erwig, 2009; Mora-Montes et al., 2012), 1 × 10^6^ macrophages in supplemented DMEM medium (Lonza, Slough, UK) were seeded onto glass based Iwaki dishes (VWR, Leistershire, UK) and cultured overnight at 37 °C with 5% CO_2_. During experiments, medium was replaced with supplemented CO_2_-independent medium containing 1 μM LysoTracker Red (Invitrogen, Paisley, UK). *C. albicans* were stained with 1 mg/ml FITC (Sigma, Dorset, UK) in 0.05 M carbonate–bicarbonate buffer (pH 9.6) for 10 min at 20 °C in the dark, washed three times, resuspended in PBS, and added to macrophages at a 3:1 ratio. Video microscopy was conducted at 37 °C with a DeltaVision Core microscope (Applied Precision, Washington, USA) and images were captured at 1 min intervals for 6 h using an EMCCD camera. Movies were visualised and edited using Volocity 5.0 imaging analysis software. Means and standard errors were calculated.
