# Supplementary figures and images for: Candida albicans infection inhibits macrophage cell division and proliferation
Source: Fungal Genet Biol. 2012 Sep;49(9):679–80. doi: 10.1016/j.fgb.2012.05.007 (PMC3430961; doi:10.1016/j.fgb.2012.05.007)

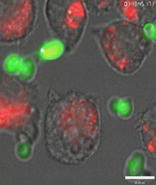

Supplement: Supplementary video 2 [file mmc2.jpg]

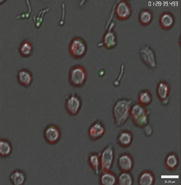

Supplement: Supplementary video 3 [file mmc3.jpg]
